# Density gradient centrifugation versus swim-up for selecting spermatozoa with improved redox activity, calcium status, and DNA integrity in an established rat model of varicocele-induced infertility

**DOI:** 10.3389/fcell.2026.1880298

**Published:** 2026-07-17

**Authors:** Ali Mutashar Naeemah Al-Hamdawee, Mazdak Razi, Ali Shalizar-Jalali, Gholamreza Najafi

**Affiliations:** 1 Division of Histology and Embryology, Department of Basic Sciences, Faculty of Veterinary Medicine, Urmia University, Urmia, Iran; 2 Division of Anatomy, Department of Basic Sciences, Faculty of Veterinary Medicine, Urmia University, Urmia, Iran

**Keywords:** DFI, DGC, redox system, spermatozoa, swim-up, varicocele

## Abstract

**Background:**

Varicocele (VCL) is a frequent pathological condition associated with impaired sperm quality, oxidative stress, altered calcium homeostasis, and sperm DNA fragmentation. The present study was not designed to validate the VCL model itself; rather, it used an established rat VCL model as a pathological source of spermatozoa to compare the efficiency of density gradient centrifugation (DGC) and swim-up (SWU) in enriching sperm populations with improved functional, redox, calcium-related, and genomic characteristics.

**Methods:**

Adult male Wistar rats were allocated to control and experimentally induced VCL groups (n = 6 animals *per* group). Two months after VCL induction, caudal epididymal spermatozoa were collected. For each animal, the initial epididymal sperm suspension was considered the stock sample; equal aliquots from the same stock suspension were then processed using SWU or DGC. Accordingly, the analytical groups were control-stock, control-SWU, control-DGC, VCL-stock, VCL-SWU, and VCL-DGC. Sperm count, motility, viability, total calcium concentration, total antioxidant capacity (TAC), total oxidant status (TOS), malondialdehyde (MDA), glutathione redox indices, protein carbonyl content, antioxidant enzyme activities, NADPH/NADP^+^ ratio, and DNA fragmentation were assessed.

**Results:**

Stock spermatozoa from VCL-induced rats showed reduced sperm count, motility, viability, TAC, glutathione-related redox capacity, and antioxidant enzyme activities, together with increased calcium concentration, TOS, MDA, protein carbonyl content, and DNA damage compared with control-stock samples. Under VCL conditions, both SWU and DGC enriched spermatozoa with improved motility and reduced DNA damage compared with VCL-stock samples. However, DGC showed greater efficiency than SWU in selecting spermatozoa with higher viability, stronger antioxidant enzyme activities, improved TAC, lower TOS, reduced lipid and protein oxidation, and lower DNA damage. The superiority of DGC over SWU was most evident in VCL-derived samples, whereas differences between the two methods were limited or absent in control-derived samples.

**Conclusion:**

In an established VCL-induced infertility model, DGC was more effective than SWU in enriching spermatozoa with improved redox activity, reduced oxidative damage, lower DNA fragmentation, and more favorable calcium status. These findings support the use of DGC as a preferable sperm preparation strategy when spermatozoa originate from a pathological oxidative-stress background such as VCL.

## Highlights

- DGC is superior to SWU in selecting DNA-preserved spermatozoa under VCL conditions.
- DGC enriches VCL-derived spermatozoa with higher antioxidant enzyme activities and improved redox balance.
- DGC reduces oxidative-damage markers more effectively than SWU in VCL-derived samples.
- Total calcium status is reduced after sperm selection but should not be interpreted as direct proof of mitochondrial calcium correction.
- The comparative advantage of DGC is more evident in pathological VCL-derived spermatozoa than in control-derived spermatozoa.

## 1 Introduction

Infertility is clinically defined as the failure to achieve pregnancy after regular unprotected sexual intercourse, and male-factor infertility contributes substantially to this condition. In many couples, assisted reproductive technology (ART) is required to support successful fertilization and pregnancy. Since the first clinical applications of ART, these techniques have contributed to millions of childbirths worldwide, and their use continues to increase (Vogiatzi et al., 2013). Under physiological *in vivo* conditions, the female reproductive tract functions as a selective environment in which motile and functionally competent spermatozoa are progressively separated from immotile spermatozoa, dead cells, leukocytes, and erythrocytes (Ali et al., 2022; Rappa et al., 2016). Therefore, sperm preparation in ART should not be viewed only as a mechanical washing step, but as a biologically relevant selection procedure intended to enrich spermatozoa with higher functional competence and lower cellular damage. Several sperm selection approaches have been developed to approximate this physiological selection process, including hyaluronic-acid binding, the ZETA method, microfluidic or related selection platforms, swim-up, and density gradient centrifugation (DGC) (Ali et al., 2022; Ghorbani-Sini et al., 2020; Kamel, 2013).

DGC separates spermatozoa on the basis of cell density by centrifuging semen or sperm suspensions through gradient media of different concentrations. Mature and morphologically normal spermatozoa generally have higher density (1.10 g/mL) than immature (1.06–1.09 g/mL), abnormal, or damaged spermatozoa, allowing DGC to enrich fractions with improved morphology and motility while reducing debris, leukocytes, and non-viable cells (Beydola et al., 2013). Previous studies have reported that DGC-prepared spermatozoa may exhibit better motility, viability, and morphology than samples processed by simpler washing approaches (Marzano et al., 2020; Vaughan and Sakkas, 2019). Nevertheless, sperm morphology and motility alone are not sufficient indicators of fertilizing competence, because sperm DNA integrity and oxidative status also critically influence embryo development and ART outcomes (Ribas-Maynou et al., 2022). Chromatin remodeling defects, oxidative damage, and impaired chromatin condensation can increase DNA denaturation and fragmentation (Osman et al., 2015; Rezaei-Agdam et al., 2019). A DNA fragmentation index (DFI) greater than approximately 30% has been associated with reduced natural and assisted reproductive success (González-Marín et al., 2012; Li et al., 2024; Liu et al., 2023). Accordingly, selecting spermatozoa with preserved DNA integrity is a central objective of sperm preparation before ART. Although several studies indicate that DGC may enrich spermatozoa with improved chromatin integrity (Ali et al., 2022; Enciso et al., 2011; Jayaraman et al., 2012; Xue et al., 2014), other reports suggest limited benefit or possible centrifugation-related adverse effects on DNA integrity under certain conditions (Stevanato et al., 2008; Yazdanpanah et al., 2021). This inconsistency highlights the need to evaluate sperm selection methods under defined pathological conditions.

Varicocele (VCL) is a major cause of male infertility and is associated with venous blood reflux, testicular hyperthermia, endocrine disruption, inflammation, oxidative stress, and sperm DNA damage. The experimental rat VCL model used in the present work has been previously established and characterized (Razi et al., 2011). Therefore, the current manuscript does not aim to revalidate or present VCL induction as its main novelty. Instead, VCL was used as a reproducible pathological model that generates spermatozoa with compromised motility, viability, redox balance, and DNA integrity. Previous work has shown that VCL may induce inflammation (Hassani-Bafrani et al., 2019), impair testicular endocrine function and apoptosis-related pathways (Dieamant et al., 2017), increase oxidative stress in testicular and semen compartments (Minas et al., 2023), and lead to sperm lipid/protein oxidation, reduced sperm count, impaired motility, disrupted chromatin compaction, and increased DNA fragmentation (Birowo et al., 2020; Jellad et al., 2021; Telli et al., 2015). These features make VCL-derived spermatozoa a suitable pathological source for testing whether sperm selection methods can preferentially enrich cells with superior functional and genomic quality.

Mitochondria are central regulators of sperm energy metabolism, reactive oxygen species (ROS) generation, calcium handling, and cell death signaling (Gualtieri et al., 2021). Mitochondrial calcium homeostasis is closely linked to respiratory-chain function and oxidant/antioxidant balance (Dridi et al., 2023; Peng and Jou, 2010). Excessive calcium accumulation can disturb mitochondrial permeability transition, decrease mitochondrial membrane potential, reduce ATP production, increase electron leakage from the electron transport chain, and promote ROS production (Hurst et al., 2017; Morciano et al., 2021; Wacquier et al., 2020). These changes may further impair total antioxidant capacity and contribute to oxidative or reductive redox imbalance (Vercesi et al., 2018; Zorov et al., 2014). However, because total calcium measurement does not directly demonstrate compartment-specific mitochondrial calcium overload, calcium-related findings must be interpreted as changes in total sperm-sample calcium status rather than as direct evidence of mitochondrial calcium flux. This distinction is important for avoiding overinterpretation and for aligning the mechanistic interpretation with the actual experimental assays performed.

Based on these considerations, the present study was designed to compare SWU and DGC as sperm preparation strategies using spermatozoa derived from both healthy control rats and an established VCL-induced infertility model. The primary objective was to determine whether DGC, compared with SWU, more effectively enriches spermatozoa with improved motility and viability, lower DNA fragmentation, better redox status, higher antioxidant enzyme activities, improved glutathione/NADPH-related redox buffering, lower oxidative damage, and more favorable total calcium status. We hypothesized that DGC would outperform SWU under VCL conditions because the density-based selection process would more efficiently separate viable, redox-competent, and DNA-intact spermatozoa from damaged sperm populations. We further hypothesized that differences between DGC and SWU would be less pronounced in control samples, where baseline sperm quality and redox balance are relatively preserved.

## 2 Materials and methods

### 2.1 Animals and ethical approval

In the present experimental original study, 12 mature male *Wistar* rats (8 weeks of age; 180–220 g) were purchased from the Animal Resource Center of Urmia University (ARCUU). After 1 week of acclimatization under standard laboratory conditions (food and water *ad libitum*, 12 h/12 h light/dark cycle, 25 °C temperature, and controlled humidity), animals were allocated to two groups: control and VCL-induced rats (n = 6 animals *per* group). The experimental protocol was reviewed and approved by the Ethics Committee of the Faculty of Veterinary Medicine, Urmia University (Permission Number: IR-UU-AEC-3/58). All experimental procedures were performed in accordance with ARRIVE guidelines (https://arriveguidelines.org), the United Kingdom. Animals (Scientific Procedures) Act 1986, and associated guidelines.

### 2.2 Induction of the established VCL model

Experimental left-sided VCL was induced according to the previously established protocol (Razi et al., 2011). Briefly, rats were anesthetized with ketamine (40 mg/kg; VOLZA, Netherlands) and xylazine (5 mg/kg; VOLZA, Netherlands). The left renal vein was partially ligated medial to the junction of the adrenal and spermatic veins, and vascular anastomotic branches between the left testicular vein and the left common iliac vein were ligated. Control animals were maintained under identical housing conditions but did not undergo VCL surgery. The VCL procedure was used to generate a pathological sperm source for subsequent comparison of sperm selection methods, not as the principal experimental endpoint of the study.

### 2.3 Experimental workflow, sample allocation, and analytical groups

The experimental workflow consisted of four sequential steps: (i) generation of control and VCL-derived sperm sources, (ii) collection of caudal epididymal spermatozoa 2 months after VCL induction, (iii) division of each stock sperm suspension into matched aliquots for SWU and DGC processing, and (iv) comparative assessment of sperm functional, redox, calcium-related, oxidative-damage, and DNA-integrity indices. For each animal, the initial epididymal sperm suspension was defined as the stock sample. From the same stock suspension, equal aliquots containing 10 × 10^6 spermatozoa were assigned to SWU or DGC. Accordingly, six analytical sample categories were generated: control-stock, control-SWU, control-DGC, VCL-stock, VCL-SWU, and VCL-DGC. Because SWU and DGC aliquots originated from the corresponding stock suspension of the same animal, comparisons were interpreted as method-dependent selection effects within each biological replicate.

### 2.4 Sperm collection, count, motility, and viability assessment

The caudal epididymis was carefully dissected under a stereo-zoom microscope (Model TL2, Olympus Co., Tokyo, Japan), separated, minced in 2 mL of Ham’s F10 medium, and incubated at 37 °C in 5% CO2 for 20 min. After incubation, epididymal tissue fragments were removed, and the released sperm suspension was used as the stock sample. Sperm count was determined in stock samples using a standard hemocytometer. Total sperm motility was assessed microscopically at 37 °C and expressed as the percentage of motile spermatozoa. Viability was evaluated using eosin-nigrosin staining; spermatozoa with red-stained heads were considered non-viable, and unstained spermatozoa were considered viable. For each sample, the percentage of viable spermatozoa was calculated and compared among the corresponding stock, SWU, and DGC groups (Razi et al., 2011). For eosin–nigrosin staining, two smears were prepared from each sample, and at least 200 spermatozoa *per* sample were evaluated under a light microscope by an observer blinded to the experimental groups. For the MTT assay, sperm suspensions from stock, SWU-selected, and DGC-selected fractions were adjusted to an equal sperm concentration before analysis. Equal aliquots of each sperm suspension were incubated with MTT solution [3-(4,5-dimethylthiazol-2-yl)-2,5-diphenyltetrazolium bromide] at 37 °C in 5% CO_2_ under light-protected conditions. After incubation, the samples were centrifuged, the supernatant was carefully removed, and the resulting formazan crystals were dissolved in dimethyl sulfoxide (DMSO). Absorbance was measured at 570 nm using a microplate reader. Blank wells containing medium, MTT reagent, and DMSO without spermatozoa were used for background correction. The MTT results were expressed as optical density values normalized to sperm number or as a percentage of the corresponding control-stock value. Because MTT reduction reflects mitochondrial dehydrogenase-dependent metabolic activity, the results were interpreted as an index of metabolically active spermatozoa rather than as a direct morphological live/dead count.

### 2.5 Sperm washing and swim-up preparation

For SWU preparation, sperm suspensions were centrifuged at 300 *g* for 10 min (Henning et al., 2015; Henning et al., 2012). This centrifugation condition was selected to limit centrifugation-related stress during sample preparation. The supernatant was discarded, and the sperm pellet was resuspended in 2 mL of fresh Ham’s F10 medium. This washing step was repeated twice to remove debris and non-sperm cells. The tubes were then incubated at 37 °C for 20 min, and spermatozoa migrating into the upper fraction were collected as the SWU-selected sample. Finally, 10 × 10^6^ spermatozoa from each SWU fraction were used for downstream analyses.

### 2.6 Density gradient centrifugation (DGC)

DGC was performed using a two-layer isotonic Percoll gradient consisting of 40% and 80% solutions (Sigma-Aldrich, United States of America). In a 15 mL conical tube, 2 mL of 80% Percoll was placed at the bottom, and 2 mL of 40% Percoll was carefully layered above it. The sperm suspension was then layered over the 40% gradient and centrifuged at 400 *g* for 20 min at room temperature. The sperm fraction located at the interface between the 40% and 80% layers was collected using a sterile pipette, transferred to a new tube, and washed twice with 5 mL of Ham’s F10 medium at 300 × g for 10 min to remove residual Percoll. The final pellet was resuspended in Ham’s F10 medium, and 10 × 10^6^ spermatozoa from each DGC-selected fraction were used for downstream analyses.

### 2.7 Acridine-orange staining for sperm DNA damage

Air-dried sperm smears were fixed in Carnoy’s solution containing one-part glacial acetic acid and three parts methanol. Acridine orange (AO) staining solution was prepared at 0.19 mg/mL, and slides were stained with freshly prepared AO in the dark for 5 min. The slides were then rinsed with washing buffer and covered with coverslips. Spermatozoa were examined under a fluorescence microscope (Canada Smart Tech, Serial No. 1706439) using an excitation wavelength of 450–490 nm. Spermatozoa with intact double-stranded DNA showed green fluorescence, whereas spermatozoa with denatured or fragmented DNA appeared yellow-orange or red. A total of 100 spermatozoa per slide were evaluated, and the percentage of spermatozoa with DNA damage was recorded for each sample.

### 2.8 Total calcium assessment

Total calcium concentration was measured using a colorimetric assay kit (Elabscience, United States; Cat No. E-BC-K103-M). Briefly, 50 µL of each sample supernatant was added to a 96-well microplate, followed by 90 µL of chromogenic reagent. The reaction was initiated by adding 60 µL of calcium assay buffer. After incubation at room temperature for 10 min, absorbance was measured at 610 nm using a microplate reader (DANA 3200, Iran). A standard curve (y = 0.12068x - 0.00198; R2 = 0.94) was generated using known calcium standards. Calcium concentration was calculated according to the kit formula (ΔA610 - b)/a × f/Cpr, where f is the dilution factor, Cpr is the protein concentration of the sample, and ΔA610 is the absolute optical density of the sample minus the blank. Values were normalized to protein content and expressed as total calcium status of the sperm sample, not as a direct measure of mitochondrial calcium overload.

### 2.9 Preparation of sperm lysates for redox and oxidative-damage assays

To assess oxidant/antioxidant indices, redox buffering, antioxidant enzyme activities, and oxidative-damage markers, spermatozoa were homogenized in appropriate assay-compatible buffer using a homogenizer (Next Avance, United States). Homogenates were centrifuged at 10,000 × g for 10 min, and supernatants were collected for biochemical analyses. Total protein concentration was determined for normalization of assays requiring protein-adjusted expression. Importantly, GPX, GR, SOD, and catalase were assessed using activity-based colorimetric assays according to the manufacturers’ protocols; therefore, these measurements represent enzymatic activity or kit-defined functional activity units rather than protein abundance or expression level.

### 2.10 Total antioxidant capacity (TAC)

TAC was determined using a commercially available assay kit (Naxifer, Navand Salamat, Iran; Cat No. NS-15013). The assay is based on the ability of antioxidants in the sample to inhibit ABTS^+^ oxidation. Briefly, 500 µL of ABTS + working solution was mixed with 100 µL of sample supernatant. After 5 min incubation at room temperature, absorbance was measured at 414 nm. TAC was calculated using the standard curve equation (y = −0.0076x + 0.6505; *R* ^2^ = 0.97) and normalized to protein content where applicable.

### 2.11 Malondialdehyde (MDA) assay

MDA was measured using a commercially available assay kit (Nalondi, Navand Salamat, Iran; Cat No. NS-15023) as an index of lipid peroxidation. The assay is based on formation of an MDA-thiobarbituric acid adduct. Briefly, 100 µL of sample supernatant was mixed with trichloroacetic acid to precipitate proteins and heated at 95 °C. After centrifugation, the supernatant was mixed with thiobarbituric acid reagent, reheated, and centrifuged. Absorbance was recorded at 532 nm. MDA values were calculated from the calibration curve (y = 0.0432x + 0.0135; *R* ^2^ = 0.93) and expressed according to the manufacturer’s instructions after appropriate normalization.

### 2.12 Total oxidant status (TOS)

TOS was measured using a commercially available assay kit (Navand Salamat, Iran, Cat N: NS-15023) according to the manufacturer’s protocol. Briefly, 30 µL of sample supernatant was added to 210 µL of assay reagent. After 20 min incubation, absorbance was read at 530 nm. TOS values were calculated using the standard curve equation (y = 0.0279x + 0.3432; *R* ^2^ = 0.96) and expressed according to the kit instructions.

### 2.13 Glutathione

Reduced glutathione (GSH) was measured using a commercial assay kit (NarGul, Navand Salamat, Iran; Cat No. NS-15087). A DTNB-based colorimetric method was employed. Briefly, 20 µL of sample supernatant was added to each well containing 20 µL of GSH buffer, followed by 120 µL of working reagent. Absorbance was recorded at 412 nm. GSH concentrations were calculated from the standard curve equation (y = 0.1789x + 0.5932; *R* ^2^ = 0.98) and expressed relative to protein content.

### 2.14 Glutathione disulfide

Glutathione disulfide (GSSG) was measured using a commercial assay kit (Navand Salamat, Iran; Cat No. NS-15085). According to the manufacturer’s protocol, 25 µL of sample supernatant was added to each well containing 25 µL of distilled water, followed by 150 µL of prepared working reagent. Absorbance was measured at 412 nm, and GSSG concentration was calculated using the standard curve equation (y = 0.9011x + 0.1856; *R* ^2^ = 0.97) and expressed relative to protein content.

### 2.15 Glutathione peroxidase activity

Glutathione peroxidase (GPX) activity was measured using a commercial activity assay kit (Navand Salamat, Iran; Cat No. NS-15083). Briefly, 40 µL of sample supernatant or standard solution was added to the microwells, followed by sequential addition of the kit reagents. Absorbance was monitored at 412 nm over four time points recorded at 15 min intervals. GPX activity was calculated using the standard curve equation (y = 0.0083x + 0.06; *R* ^2^ = 0.94) and expressed as the kit-defined enzymatic activity unit.

### 2.16 Glutathione reductase activity

Glutathione reductase (GR) activity was measured using a commercial activity assay kit (NarGul, Navand Salamat, Iran; Cat No. NS-15085). Equal volumes of the prepared DTNB solution and GR reaction reagent were mixed, and 120 µL of the mixture was added to the microwells. After 1 min incubation in the dark, 60 µL of cofactor solution was added. Absorbance was immediately measured at 412 nm. GR activity was calculated according to the kit protocol using the standard curve equation (y = 0.1789x + 0.5932; *R* ^2^ = 0.96) and expressed as the kit-defined enzymatic activity unit normalized where applicable.

### 2.17 Superoxide dismutase activity

Superoxide dismutase (SOD) activity was determined using the corresponding commercial colorimetric activity assay kit (Nasdox, Navand Salamat, Iran; Cat No. NS-15085) according to the manufacturer’s instructions. The assay was performed on sperm lysate supernatants, and absorbance was recorded at the wavelength recommended by the kit. SOD results were interpreted as enzymatic activity and not as SOD protein expression or abundance.

### 2.18 Catalase activity

Catalase (CAT) activity was measured using a commercially available activity assay kit (Nactaz, Navand Salamat, Iran; Cat No. NS-15054). For activity determination, 60 µL of sample supernatant was mixed with 240 µL of kit reagents in a final volume of 300 µL. Absorbance was recorded at 550 nm immediately after completion of the reaction steps. CAT activity was calculated using the standard curve equation (y = 0.0087x + 0.3244; *R* ^2^ = 0.96) and expressed according to the manufacturer’s instructions after normalization where applicable.

### 2.19 NADPH/NADP^+^ assay

Intracellular NADPH and NADP + levels were quantified using a commercial NADPH/NADP + assay kit (Abcam, UK; Cat No. ab65349), following the manufacturer’s protocol. Briefly, 100 µL of sperm extract was combined with 50 µL of assay buffer in a 96-well microplate. For NADPH measurement, 50 µL of NADPH detection reagent was added, and the plate was incubated at 37 °C for 30 min. After completion of NADPH measurement, 50 µL of NADP + detection reagent was added to each well, followed by a second 30 min incubation at 37 °C. Absorbance was recorded at 450 nm using a microplate reader (DANA 3200). NADPH and NADP^+^ concentrations were calculated from standard curves provided with the kit, and the NADPH/NADP + ratio was used as an index of redox buffering capacity.

### 2.20 Protein carbonyl assessment

Protein carbonyl content was measured using a commercial assay kit (Elabscience, United States; Cat No. E-BC-K117-S). Briefly, 0.1 mL of sample supernatant was mixed with 0.4 mL of 2,4-dinitrophenylhydrazine solution. Control tubes were prepared using acid reagent instead of DNPH. Samples were incubated for 30 min at 37 °C in the dark, precipitated, centrifuged at 13,780 × g for 10 min at 4 °C, and washed three times with an anhydrous ethanol-ethyl acetate mixture. The final pellet was dissolved in denaturant, incubated at 37 °C for 15 min, and centrifuged at 13,780 × g for 15 min at 4 °C. Absorbance of the supernatant was measured at 370 nm using a quartz cuvette, and protein carbonyl content was calculated according to the manufacturer’s formula.

### 2.21 Statistical analysis

Statistical analyses were performed using GraphPad Prism software (version 9, San Diego, CA, United States of America). Data were assessed for normality and homogeneity of variance before inferential analysis. Multiple-group comparisons were performed using one-way analysis of variance followed by Tukey’s multiple comparison test. The statistical interpretation was structured to address the experimental design: first, control-stock and VCL-stock samples were compared to confirm the pathological sperm profile generated by VCL; second, within each condition, SWU and DGC were compared with the corresponding stock sample to evaluate selection effects; third, DGC and SWU were compared directly to determine method-specific superiority; and fourth, VCL-derived samples were compared with the corresponding control-derived samples processed by the same method when biologically relevant. Results are presented as mean ± SD. A p-value less than 0.05 was considered statistically significant, and exact p-values are reported where available.

## 3 Results

### 3.1 Confirmation of the pathological sperm profile and selection effects on motility and viability

**FIGURE 1:**
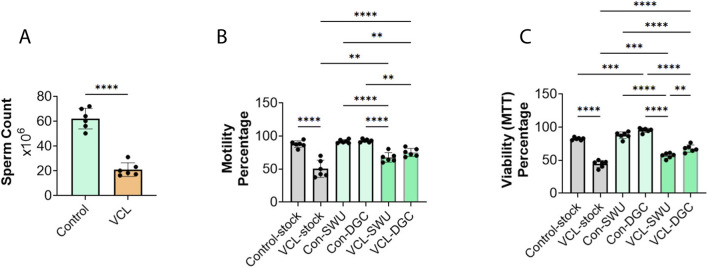
Mean changes in spermatozoa **(A)** count **(B)** motility, and **(C)** MTT-based metabolic viability in control-stock and VCL-stock samples and after SWU and DGC processing where applicable. Data are presented as mean ± SD. VCL: varicocele; SWU: swim-up; DGC: density gradient centrifugation.

Sperm count, motility, and viability were first assessed to verify that the established VCL model generated the expected pathological sperm profile. This analysis was used as model confirmation and not as the primary novelty of the study. Compared with control-stock samples, VCL-stock samples showed significantly reduced sperm count (p = 0.0001), motility (p = 0.0001), and viability (p = 0.0001), confirming the presence of impaired sperm quality in the VCL-derived stock suspension (Figures 1A–C).

The selection efficiency of SWU and DGC was then evaluated within control and VCL-derived samples. In the VCL group, both SWU (p = 0.0001) and DGC (p = 0.0001) selected spermatozoa with significantly higher motility than VCL-stock samples. In control-derived samples, motility did not significantly differ among stock, SWU, and DGC fractions, indicating that selection-related improvement was limited when baseline motility was already preserved. Direct comparison between SWU and DGC showed no significant difference in motility within either control or VCL-derived samples.

For viability, DGC significantly enriched viable spermatozoa compared with stock samples, particularly under VCL conditions (p = 0.0001). In the control group, SWU did not significantly increase viability compared with control-stock samples. In contrast, under VCL conditions, DGC yielded significantly higher sperm viability than SWU (p = 0.02). Nevertheless, even after SWU and DGC processing, VCL-derived fractions retained lower viability than their corresponding control-derived fractions, indicating that sperm selection improved but did not completely normalize the VCL-associated viability deficit.

### 3.2 Total calcium status

**FIGURE 2:**
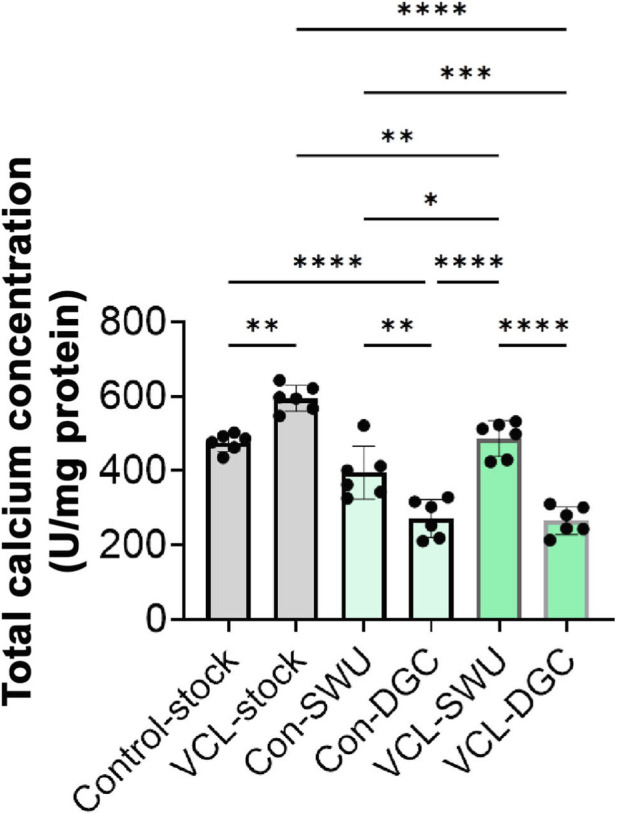
Mean changes in total calcium concentration/status in stock, SWU-selected, and DGC-selected sperm fractions. Values were normalized to total protein content and expressed as U/mg protein. Data are presented as mean ± SD. VCL: varicocele; SWU: swim-up; DGC: density gradient centrifugation.

Total calcium concentration was significantly higher in VCL-stock samples than in control-stock samples (p = 0.001), indicating altered calcium status in spermatozoa derived from the VCL model. Both SWU and DGC reduced total calcium concentration compared with the corresponding stock samples. In the control group, SWU (p = 0.05) and DGC (p = 0.0001) significantly decreased calcium levels relative to control-stock samples. A similar reduction was observed in VCL-derived samples after SWU and DGC processing. However, despite this reduction, VCL-SWU and VCL-DGC samples still exhibited significantly higher calcium concentrations than their corresponding control-SWU and control-DGC samples (p = 0.0001 for both comparisons; Figure 2). These findings indicate that both methods enriched fractions with lower total calcium status, but selection did not completely eliminate the VCL-associated calcium abnormality.

### 3.3 Antioxidant enzyme activities

Antioxidant enzyme activities were assessed to determine whether SWU and DGC differentially enriched spermatozoa with functional antioxidant defense. Compared with control-stock samples, VCL-stock samples showed significantly lower GPX (p = 0.02), GR (p = 0.01), SOD (p = 0.008), and CAT (p = 0.03) activities, confirming suppression of enzymatic antioxidant defense under VCL conditions.

**FIGURE 3:**
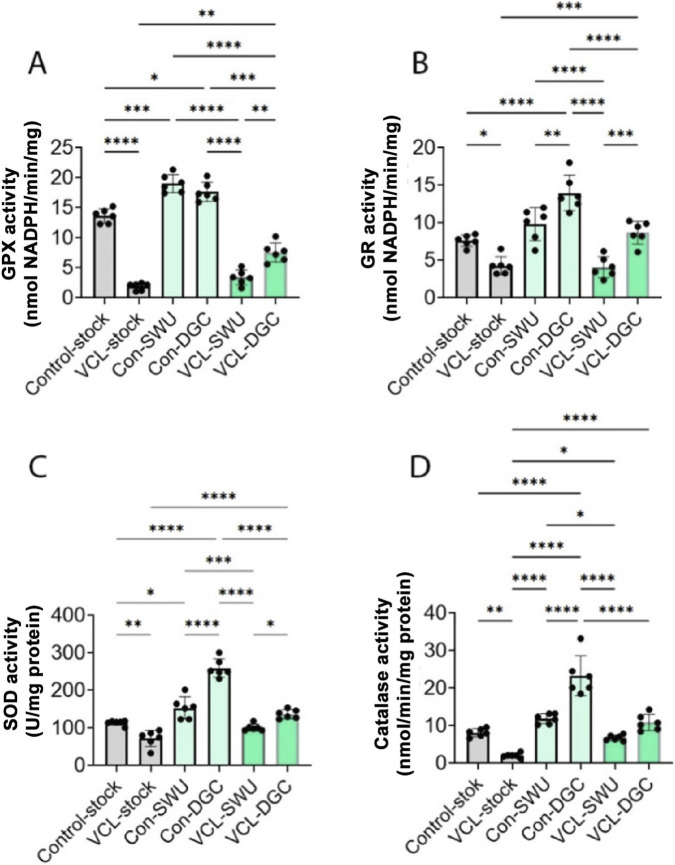
Mean changes in antioxidant enzyme activities, including **(A)** GPX **(B)** GR **(C)** SOD, and **(D)** catalase, in stock, SWU-selected, and DGC-selected sperm samples. Enzyme activities were determined using activity-based colorimetric assays and normalized to total protein content. Data are presented as mean ± SD. VCL: varicocele; SWU: swim-up; DGC: density gradient centrifugation.

In control-derived samples, SWU significantly increased GPX (p = 0.001), GR (p = 0.01), and CAT (p < 0.0001) activity compared with control-stock samples. In VCL-derived samples, SWU significantly increased GPX (p = 0.009) and CAT (p = 0.02) activity, whereas SOD activity did not significantly change after SWU (p = 0.90). DGC significantly increased GPX, GR, SOD, and CAT activities compared with the corresponding stock samples in both control and VCL groups, with particularly strong effects in VCL-derived samples (GPX, GR, SOD, and CAT: p = 0.0001 where indicated). Direct comparison between selection methods demonstrated that DGC was more effective than SWU in enriching spermatozoa with higher GPX (control: p = 0.01; VCL: p = 0.004), GR (control: p = 0.006; VCL: p = 0.002), SOD (control: p = 0.0001; VCL: p = 0.031), and CAT (control: p = 0.0001; VCL: p = 0.049) activities (Figures 3A–D).

### 3.4 Correlation between total calcium status and antioxidant enzyme activities

**FIGURE 4:**
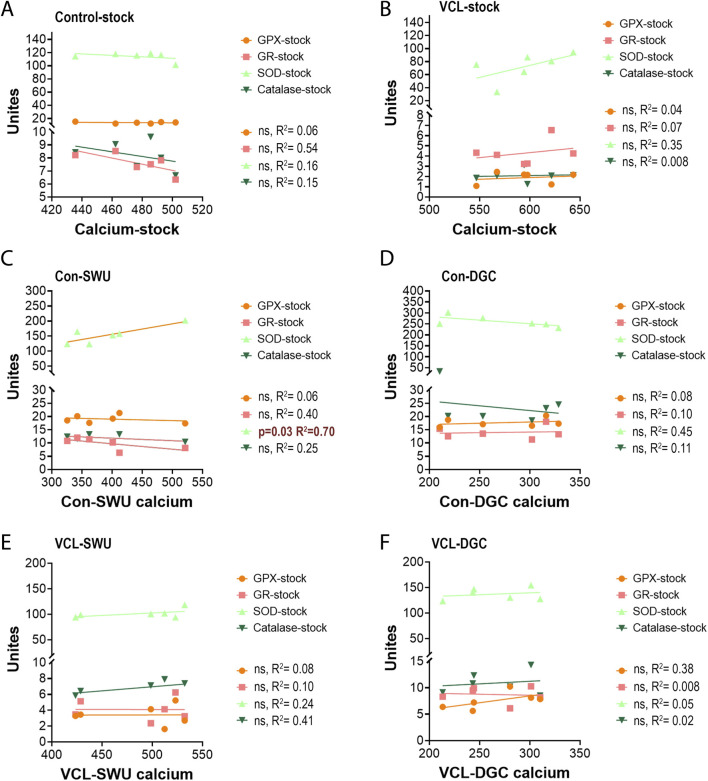
Correlation analyses between total calcium concentration and antioxidant enzyme activities in different sperm sample categories. **(A)** Control-stock, **(B)** VCL-stock, **(C)** Con-SWU, **(D)** Con-DGC, **(E)** VCL-SWU, and **(F)** VCL-DGC. Most correlations were not significant. A statistically significant positive correlation was observed only between total calcium concentration and SOD activity in the Con-SWU group (Panel C; *p* = 0.03, R^2^ = 0.70). GPX, glutathione peroxidase; GR, glutathione reductase; SOD, superoxide dismutase; CAT, catalase; ns, non-significant; VCL, varicocele; SWU, swim-up; DGC, density.

Correlation analysis was performed to determine whether total calcium status was associated with antioxidant enzyme activities before and after sperm selection. Overall, no significant correlations were observed between total calcium concentration and GPX, GR, SOD, or CAT activity in control or VCL-derived samples before or after SWU or DGC processing (p > 0.05). The only exception was a significant correlation between calcium concentration and SOD activity after SWU in the control group (p = 0.03; Figures 4A–F). Therefore, although VCL altered both calcium status and enzymatic antioxidant activity, the present data do not support a direct linear association between total calcium concentration and antioxidant enzyme activities across all sample categories.

### 3.5 Glutathione redox indices and NADPH/NADP^+^ ratio

**FIGURE 5:**
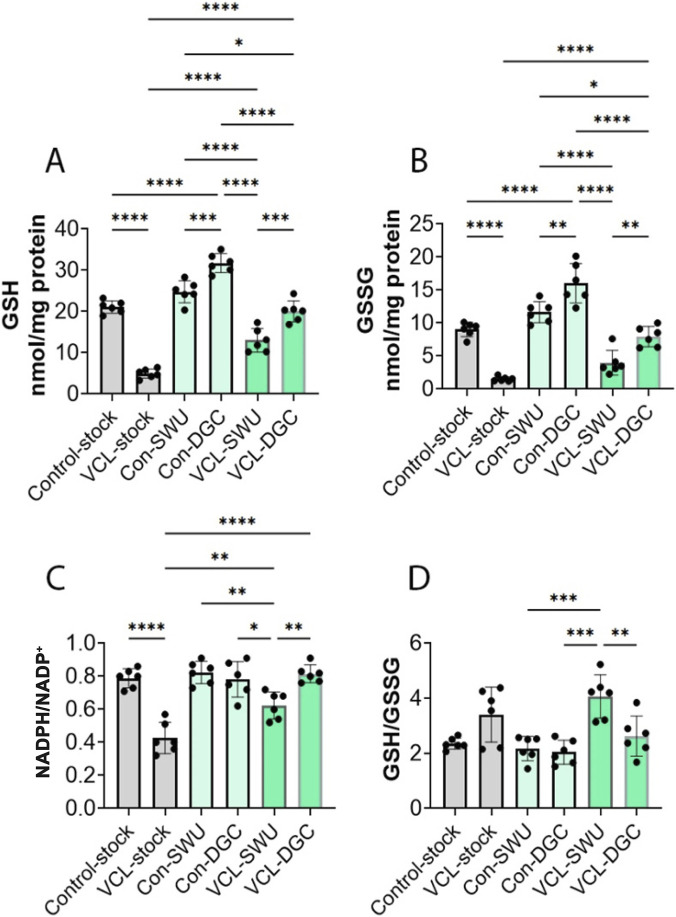
Mean changes in **(A)** reduced glutathione (GSH) **(B)** glutathione disulfide (GSSG) **(C)** NADPH/NADP^+^ ratio, and **(D)** GSH/GSSG ratio in stock, SWU-selected, and DGC-selected sperm samples. Data are presented as mean ± SD. VCL: varicocele; SWU: swim-up; DGC: density gradient centrifugation.

VCL-stock samples exhibited significantly lower GSH (p = 0.001) and GSSG (p = 0.0001) levels compared with control-stock samples, indicating disruption of glutathione-associated redox buffering under VCL conditions. DGC significantly enriched spermatozoa with higher GSH (p = 0.0001) and GSSG (p = 0.001) levels compared with the corresponding stock samples. By contrast, SWU did not significantly change GSH or GSSG levels compared with stock samples in either control or VCL conditions (p > 0.05). Direct comparison between SWU and DGC showed that DGC was more effective in selecting spermatozoa with higher GSH and GSSG levels in both control (GSH and GSSG: p = 0.0001) and VCL-derived samples (GSH: p = 0.0009; GSSG: p = 0.008). Despite these improvements, GSH and GSSG levels remained significantly lower in VCL-derived fractions than in the corresponding control-derived fractions (p = 0.0001; Figures 5A,B).

The NADPH/NADP^+^ ratio was significantly decreased in VCL-stock samples compared with control-stock samples (p = 0.0001), whereas the GSH/GSSG ratio showed a non-significant increase (p > 0.05). SWU did not significantly affect NADPH/NADP^+^ or GSH/GSSG ratios in control samples. In VCL-derived samples, both SWU and DGC increased the NADPH/NADP^+^ ratio compared with VCL-stock samples, while the GSH/GSSG ratio was not markedly changed after either selection method. Direct comparison between SWU and DGC revealed no significant difference in control-derived samples; however, in VCL-derived samples, DGC significantly increased the NADPH/NADP^+^ ratio (p = 0.02) and decreased the GSH/GSSG ratio (p = 0.04) compared with SWU (Figures 5C,D).

### 3.6 Total antioxidant capacity and total oxidant status

**FIGURE 6:**
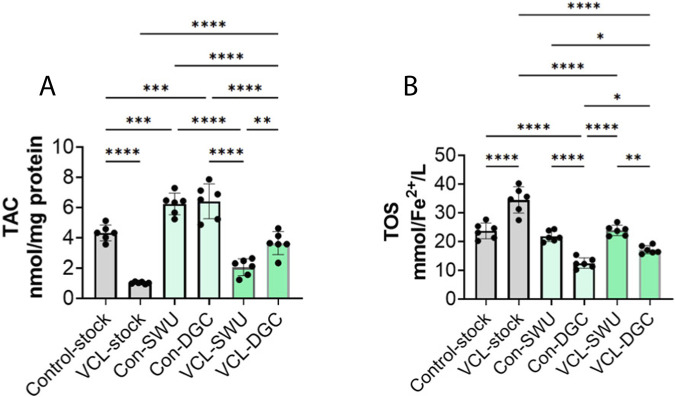
Mean changes in **(A)** total antioxidant capacity (TAC) and **(B)** total oxidant status (TOS) in stock, SWU-selected, and DGC-selected sperm samples. Data are presented as mean ± SD. VCL: varicocele; SWU: swim-up; DGC: density gradient centrifugation.

VCL-stock samples showed significantly lower TAC (p = 0.0001) and higher TOS (p = 0.0001) than control-stock samples, confirming a shift toward oxidative stress under VCL conditions. In control-derived samples, both SWU (p = 0.007) and DGC (p = 0.002) increased TAC compared with control-stock samples. In VCL-derived samples, SWU did not significantly improve TAC compared with VCL-stock samples (p > 0.05), whereas DGC significantly increased TAC (p = 0.001). Direct comparison between methods showed no significant difference between SWU and DGC in control-derived samples. Under VCL conditions, however, DGC selected spermatozoa with significantly higher TAC than SWU (p = 0.006; Figure 6A).

For TOS, DGC significantly reduced oxidant status compared with stock samples in both control (p = 0.0001) and VCL-derived (p = 0.0001) groups. SWU did not significantly alter TOS in control-derived samples (p > 0.05). Direct comparison between selection methods demonstrated that DGC produced significantly lower TOS values than SWU in both control (p = 0.0001) and VCL-derived (p = 0.001) samples (Figure 6B). These findings indicate that DGC had a stronger effect than SWU in enriching sperm populations with improved oxidant/antioxidant balance, especially under VCL conditions.

### 3.7 Lipid and protein oxidative damage

**FIGURE 7:**
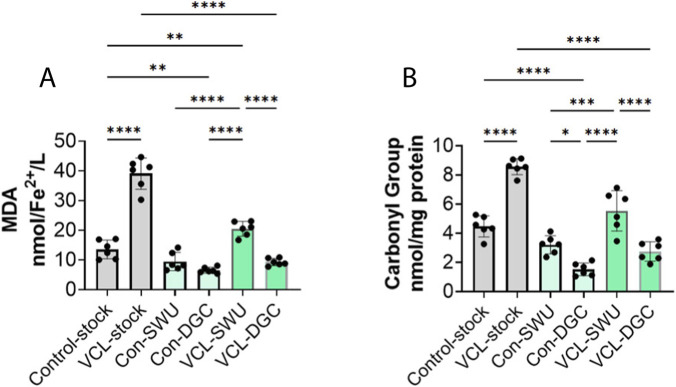
Mean changes in **(A)** malondialdehyde (MDA) and **(B)** protein carbonyl content in stock, SWU-selected, and DGC-selected sperm samples. Data are presented as mean ± SD. VCL: varicocele; SWU: swim-up; DGC: density gradient centrifugation.

MDA and protein carbonyl content were assessed as markers of lipid and protein oxidation, respectively. VCL-stock samples showed significantly higher MDA (p = 0.0001) and protein carbonyl levels (p = 0.0001) than control-stock samples. DGC significantly decreased MDA levels compared with stock samples in both control (p = 0.004) and VCL-derived (p = 0.0001) groups. In control-derived samples, SWU did not significantly change MDA compared with stock samples (p > 0.05). In VCL-derived samples, DGC reduced MDA more effectively than SWU, and direct comparison confirmed significantly lower MDA after DGC than after SWU under VCL conditions (Figure 7A).

Protein carbonyl content was significantly reduced by both SWU and DGC compared with VCL-stock samples (p = 0.0001), indicating that both methods enriched fractions with lower protein oxidation under pathological conditions. In control-derived samples, SWU did not significantly change carbonyl content (p > 0.05), whereas DGC significantly reduced carbonyl content compared with control-stock samples (p = 0.0001). Direct comparison showed no significant difference between SWU and DGC in control-derived samples, but DGC reduced carbonyl content more effectively than SWU in VCL-derived samples (p = 0.0001; Figure 7B).

### 3.8 Correlations between total calcium status, antioxidant capacity, and oxidative-damage markers

**FIGURE 8:**
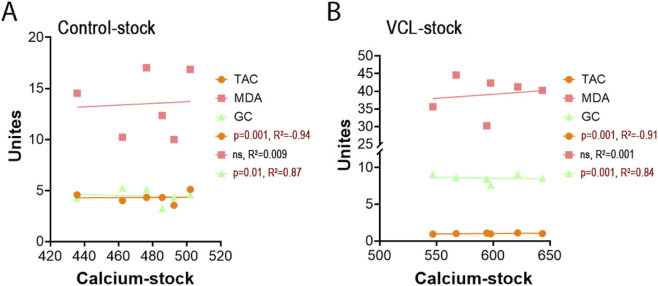
Correlation analyses between total calcium concentration and antioxidant capacity, lipid peroxidation, and protein oxidation markers in stock sperm samples. **(A)** Control-stock samples. **(B)** VCL-stock samples. Total calcium concentration was positively correlated with total antioxidant capacity (TAC) and (GC), whereas no significant correlation was observed between total calcium concentration and malondialdehyde (MDA). TAC, total antioxidant capacity; MDA, malondialdehyde; GC, carbonyl groups.

Correlation analysis was performed to clarify the relationship between total calcium status, TAC, lipid peroxidation, and protein oxidation in stock samples. Total calcium concentration was negatively correlated with TAC, whereas positive associations were observed between total calcium concentration and oxidative-damage markers, particularly MDA and protein carbonyl content (Figures 8A,B). These findings suggest that increased total calcium status is more closely aligned with global oxidative damage and reduced antioxidant capacity than with individual antioxidant enzyme activities.

### 3.9 Sperm DNA damage

**FIGURE 9:**
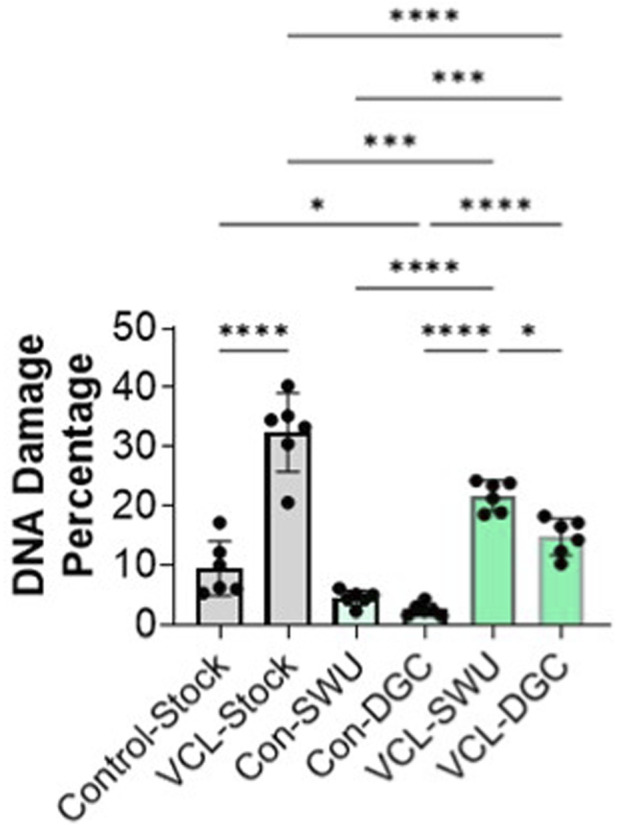
Mean changes in the percentage of spermatozoa with DNA damage in stock, SWU-selected, and DGC-selected sperm samples. Data are presented as mean ± SD. VCL: varicocele; SWU: swim-up; DGC: density gradient centrifugation.

The percentage of spermatozoa with DNA damage was significantly higher in VCL-stock samples than in control-stock samples (p = 0.0001), confirming the DNA-damaging effect of the VCL pathological condition. In control-derived samples, SWU did not significantly reduce DNA damage compared with control-stock samples (p > 0.05), whereas DGC significantly reduced the percentage of spermatozoa with DNA damage (p = 0.002). Under VCL conditions, both SWU (p = 0.0001) and DGC (p = 0.0001) significantly reduced DNA damage compared with VCL-stock samples. Direct comparison between selection methods showed no significant difference between SWU and DGC in control-derived samples (p > 0.05). However, in VCL-derived samples, DGC selected spermatozoa with significantly lower DNA damage than SWU (p = 0.03; Figure 9). These results identify DNA integrity as a major endpoint for which DGC provided superior selection efficiency under VCL-associated oxidative-stress conditions.

## 4 Discussion

The present study was designed to compare DGC and SWU as sperm selection methods using spermatozoa obtained from healthy controls and from an established VCL-induced infertility model. Importantly, the objective was not to validate the VCL model, which has been previously characterized (Razi et al., 2011), but to use VCL-derived spermatozoa as a pathological source with oxidative stress, altered calcium status, reduced antioxidant defense, and increased DNA damage. The main finding was that DGC was more efficient than SWU in enriching VCL-derived spermatozoa with higher viability, stronger antioxidant enzyme activities, improved TAC, reduced TOS, lower lipid and protein oxidation, and reduced DNA damage. In contrast, differences between SWU and DGC were generally limited in control-derived samples, where baseline sperm function and redox balance were comparatively preserved. This pattern supports the hypothesis that density-based selection provides its greatest advantage when the original sperm population is heterogeneous and pathologically compromised.

Sperm selection in ART is primarily intended to enrich the population of spermatozoa with optimal fertilizing potential. Although motility and morphology are conventional selection indicators, DNA integrity is equally critical because paternal genomic damage may compromise fertilization, embryo development, and reproductive outcomes (González-Marín et al., 2012; Ribas-Maynou et al., 2022). Spermatozoa with limited DNA damage may be partially repaired by oocyte repair mechanisms, whereas extensive DNA fragmentation can exceed the reparative capacity of the oocyte and impair embryo viability (Leem et al., 2024; Marinaro and Schlegel, 2023). A DFI exceeding approximately 30% is associated with reduced natural and assisted reproductive success (González-Marín et al., 2012; Li et al., 2024; Liu et al., 2023). In this context, the current findings demonstrate that both SWU and DGC reduced DNA damage in VCL-derived samples, but DGC produced a greater reduction than SWU. These results are consistent with reports indicating that DGC can remove spermatozoa with DNA damage or poorly condensed chromatin (Ali et al., 2022; Enciso et al., 2011; Jayaraman et al., 2012; Xue et al., 2014), while also acknowledging that the effect of DGC may vary depending on sample condition and processing stress (Stevanato et al., 2008; Yazdanpanah et al., 2021).

The superiority of DGC under VCL conditions may be explained, at least in part, by its ability to select spermatozoa with improved redox function. Spermatozoa are highly vulnerable to oxidative damage because their plasma membrane contains abundant polyunsaturated fatty acids and because their cytoplasmic antioxidant reserve is limited (Agarwal et al., 2019; Khosravanian et al., 2014; Razi et al., 2011). Under VCL conditions, excessive ROS generation and insufficient antioxidant defense can lead to lipid peroxidation, protein oxidation, and DNA fragmentation (Minas et al., 2023; Xiao and Loscalzo, 2020). Consistent with this framework, VCL-stock samples in the present study showed lower TAC and higher TOS, MDA, and protein carbonyl content than control-stock samples. DGC, more than SWU, selected spermatozoa with higher TAC and lower TOS, MDA, and carbonyl content, indicating a stronger capacity to enrich sperm fractions with lower oxidative injury. Because oxidative damage is a major driver of sperm DNA fragmentation, the lower DNA damage observed after DGC is biologically consistent with the improved oxidant/antioxidant balance observed in DGC-selected VCL samples.

A key methodological concern raised by the reviewer was whether antioxidant enzymes were assessed as protein abundance or as functional enzymatic activity. The revised Methods clarify that GPX, GR, SOD, and CAT were evaluated using activity-based colorimetric assays and interpreted as enzymatic activity or kit-defined functional activity units, not as expression levels or protein abundance. This distinction is critical because protein abundance alone does not necessarily reflect catalytic antioxidant capacity. The results showed that VCL reduced GPX, GR, SOD, and CAT activities, whereas DGC significantly enriched spermatozoa with higher activities of these enzymes compared with SWU. These findings indicate that DGC selection was associated with functional antioxidant competence, not merely with altered protein presence. In addition, the improved NADPH/NADP^+^ and glutathione-related indices after DGC suggest that DGC-selected spermatozoa had better redox buffering capacity. Redox balance is maintained by integrated interactions among enzymatic antioxidant defense, glutathione cycling, NADPH availability, and oxidant production (Benedetti et al., 2012; Checa and Aran, 2020; Radmanesh et al., 2021; Xiao and Loscalzo, 2020). Therefore, evaluating TAC or TOS alone would not sufficiently characterize the redox status of selected spermatozoa; the combined assessment of enzyme activities, glutathione balance, NADPH/NADP^+^ ratio, and oxidative damage provides a more coherent interpretation.

**FIGURE 10:**
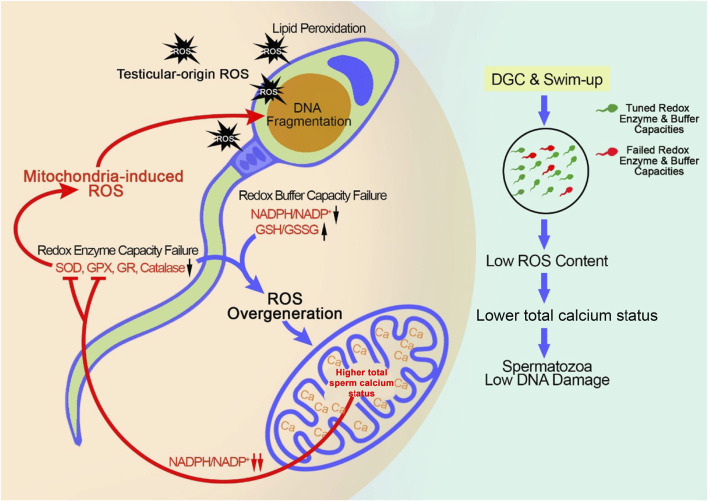
Proposed interpretative model for the relationship among VCL-associated oxidative stress, altered total calcium status, redox imbalance, and sperm DNA damage, and the potential selection advantage of DGC over SWU. Under VCL conditions, spermatozoa are exposed to oxidative stress, reduced antioxidant enzyme activity, impaired redox buffering, lipid/protein oxidation, and increased DNA damage. DGC appears to enrich sperm fractions with lower oxidative burden, improved antioxidant activity, lower total calcium status, and reduced DNA damage. Because mitochondrial calcium flux and calcium-transport mechanisms were not directly measured, the model should be interpreted as a proposed association rather than direct mechanistic proof.

The relationship between calcium status and sperm oxidative damage is complex. Mitochondria are both sources and targets of ROS, and mitochondrial calcium handling is closely linked to respiratory-chain activity, ATP production, and redox signaling (Gualtieri et al., 2021; Lee et al., 2023; Peng and Jou, 2010). Excess calcium may stimulate mitochondrial dehydrogenases, alter electron transport, and contribute to ROS generation, whereas oxidative stress may impair calcium transport systems and further promote calcium accumulation (Brookes et al., 2004; Dridi et al., 2023; Hurst et al., 2017; Matuz-Mares et al., 2022; Morciano et al., 2021; Wacquier et al., 2020; Zhang et al., 2021; Zorov et al., 2014). In the present study, VCL-stock samples showed higher total calcium concentration than control-stock samples, and both SWU and DGC reduced total calcium status relative to stock samples. However, correlation analysis did not reveal a consistent linear association between total calcium concentration and antioxidant enzyme activities. Instead, total calcium status correlated more closely with TAC and oxidative-damage markers, including MDA and carbonyl content. A schematic summary of the proposed mechanisms underlying the comparative effects of density gradient centrifugation (DGC) and swim-up (SWU) on oxidative stress, calcium status, and sperm DNA integrity is presented in Figure 10. These results suggest that total calcium status in VCL-derived spermatozoa may reflect the broader oxidative-damage burden rather than directly predicting individual antioxidant enzyme activity. Because mitochondrial calcium influx, mitochondrial permeability transition, and calcium transporter activity were not directly measured, the revised interpretation avoids claiming that DGC directly corrects mitochondrial calcium overload. Rather, DGC appears to select spermatozoa with lower total calcium status and lower oxidative damage, which may indirectly indicate better mitochondrial integrity.

The observation that DGC outperformed SWU mainly in VCL-derived samples is also biologically meaningful. In control samples, spermatozoa are expected to have relatively preserved motility, redox balance, and DNA integrity; therefore, additional selection may produce only modest improvements. Under VCL conditions, however, the stock sperm population contains a larger proportion of dead, immotile, oxidatively damaged, and DNA-fragmented spermatozoa. DGC may be more effective in such heterogeneous pathological samples because it separates spermatozoa according to density and thereby more efficiently removes morphologically abnormal, damaged, and less functionally competent cells (Beydola et al., 2013; Marzano et al., 2020; Vaughan and Sakkas, 2019). This interpretation is consistent with the current finding that DGC, but not always SWU, improved multiple functional and biochemical endpoints under VCL conditions. The findings also explain why presenting VCL-induced reductions in motility and viability as the opening result could be confusing if not properly framed. In the revised manuscript, these parameters are presented as confirmation of the expected pathological sperm profile, while the primary experimental focus is the comparative selection performance of SWU and DGC.

## 5 Study limitations

Several limitations should be acknowledged. First, the study used an established experimental VCL model as a pathological sperm source. Therefore, the findings should be interpreted as evidence for method-dependent sperm selection efficiency rather than as validation of VCL pathogenesis. Second, total calcium concentration was measured in sperm samples, but mitochondrial calcium compartmentalization, calcium uniporter activity, and calcium efflux mechanisms were not directly evaluated. Thus, calcium-related interpretations should be limited to total sample calcium status. Third, although antioxidant enzyme activities were assessed using activity-based assays, direct measurement of ROS generation, mitochondrial membrane potential, ATP production, and live-cell calcium dynamics would further strengthen mechanistic conclusions. Fourth, acridine-orange staining provides useful information on DNA denaturation or damage, but additional assays such as TUNEL, comet assay, or sperm chromatin structure assay could provide complementary confirmation of DNA fragmentation. Finally, translation of these findings to clinical ART requires validation in human semen samples from patients with clinical varicocele.

## 6 Conclusion

The present study demonstrates that DGC is more effective than SWU in selecting spermatozoa with improved functional competence, stronger antioxidant enzyme activities, better redox balance, reduced oxidative damage, lower total calcium status, and preserved DNA integrity under VCL-induced pathological conditions. The VCL model was used as an established oxidative-stress sperm source, not as the primary object of validation. The superiority of DGC was most evident in VCL-derived samples, whereas both selection methods had limited additional effects in control-derived samples with preserved baseline sperm quality. Overall, DGC appears to be a preferable sperm preparation strategy when the initial sperm population is compromised by VCL-associated oxidative and genomic damage.

## Data Availability

The datasets presented in this article are not readily available because The data that support the findings of this study are available from the corresponding author upon reasonable request. Requests to access the datasets should be directed to Mazdak Razi, m.razi@urmia.ac.ir.
